# Endocervical curettage for diagnosing high-grade squamous intraepithelial lesions or worse in women with type 3 transformation zone lesions: a retrospective, observational study

**DOI:** 10.1186/s12905-023-02297-0

**Published:** 2023-05-09

**Authors:** Bingrui Wei, Qing Li, Samuel Seery, Youlin Qiao, Yu Jiang

**Affiliations:** 1 Department of Epidemiology and Biostatistics, School of Population Medicine and Public Health, Chinese Academy of Medical Sciences and Peking Union Medical College, Beijing, 100730 China; 2 Diagnosis and Treatment for Cervical Lesions Center, Shenzhen Maternity and Child Healthcare Hospital, Shenzhen, 518028 China; 3 Faculty of Health and Medicine, Division of Health Research, Lancaster University, Lancaster, LA1 4YW UK

**Keywords:** Cervical cancer, Colposcopy, Endocervical curettage, High-grade squamous intraepithelial lesion

## Abstract

**Background:**

This study aimed to assess the value of endocervical curettage (ECC) in detecting high-grade squamous intraepithelial lesion or worse (HSIL+) in women with type 3 transformation zone (TZ3) lesions, and to identify the clinical characteristics of patients with TZ3 lesions who benefit most from ECC.

**Methods:**

This retrospective, multicenter study included 1,905 women with TZ3 lesions who attended cervical screening in one of seven tertiary hospitals in China between January 2020 and November 2021. All participants had received abnormal results and had been referred to colposcopy. Risk factors were identified through univariate and multifactorial logistic analyses.

**Results:**

In total, 20.5% (*n* = 391) of HSIL+ cases with TZ3 lesions had been diagnosed with biopsy and ECC. ECC detected 0.8% (*n* = 15) HSIL+ cases otherwise missed by biopsy alone. Multivariate analysis identified four factors which influenced detection performance. The probability of detecting HSIL+ with ECC is 2.653 (95% confidence interval [CI] 1.009–6.977) times greater in women aged 40–49 years and 2.545 (95% CI 0.965–6.716) times greater for those aged 50 years and older compared to those younger than 30 years. The probability of ASC-H (atypical squamous cells, cannot exclude high-grade squamous intraepithelial lesion) and HSIL cytologies were respectively 2.415 (95% CI 1.213–4.808) and 2.933 (95% CI 1.648–5.220) times higher than for NILM (negative for intraepithelial lesion or malignancy). Women with human papillomavirus 16/18 infections were 2.299 (95% CI 0.942–5.613) times more likely to be HSIL+. Precancerous lesions were 35.884 (95% CI 12.214–105.426) times more likely in women who had high-grade colposcopic impressions compared to those with normal impressions.

**Conclusions:**

ECC should be performed for patients with ASC-H or HSIL cytologies, human papillomavirus 16/18 infections, and for those with high-grade colposcopic impressions. This will increase the number of HSIL+ cases identified using biopsy by reducing the number of false negatives.

**Supplementary Information:**

The online version contains supplementary material available at 10.1186/s12905-023-02297-0.

## Background

Cervical cancer is the sixth most common malignancy and eighth most common cause of cancer-related mortality in Chinese women, with 119,300 new cases and 59,060 deaths from cervical cancer in 2020. This represents 5% of all female cancer-related deaths in China and both indicators highlight a continuous upward trajectory over the past five years [1]. Early interventions in cervical cancer development rely on adequate, accurate, and efficient screening. The current gold standard for cervical cancer diagnosis in patients with abnormal cytologies or with human papillomavirus (HPV) is colposcopic tissue removal from lesions and histopathological testing. However, for between 2–12% of women with transformation zone type 3 (TZ3) lesions, atrophy analysis and conventional lesion-directed ectocervical biopsies fail to detect occult lesions in the cervical canal [2–4]. Undiagnosed occult lesions can progress rapidly and can have dire consequences.

At present, endocervical curettage (ECC) is generally only used in clinical practice for adjunctive biopsies when part or all of the TZ is not visible. However, in China, there are no consistent indications for screening high-risk subgroups. In fact, many Chinese clinicians perform ECC simply to avoid missing HSIL + cases, despite knowing that not all women will benefit from the procedure. The value of ECC in HSIL+ detection remains controversial, with a detection rate of only 1–9.3% observed in previous studies [4, 5]. ECC is also invasive, and repeated pulling and scraping in the cervical canal can be painful for patients and reduce the likelihood of attending follow-up appointments [6–8]. Additionally, specimen cosistency obtained by ECC is only moderate (κ = 0.52) [9] and interobserver agreement is poor [10]. Numerous studies have also found that CIN2+ is more prevalent in women with TZ3 lesions, compared to those without (8–27% vs 1.3–12%) however, this is only when colposcopy is satisfactory [9, 11, 12]. The aim of this study was to determine whether the detection performance and ECC accuracy are sufficiently high for TZ3 lesion cases to outweigh the disadvantages.

## Methods

### Study design

Data were retrospectively collected from electronic medical records of women who underwent colposcopic examination with ECC and biopsy for abnormal cervical screening results at any of seven tertiary hospitals in mainland China. This sample was collected from January 2020 and to November 2021. Abnormal screenings were defined according to positive HPV test results and/or with positive cytologies. Only patients who had received HPV testing, cytology, colposcopy, biopsy, or ECC were enrolled.

Those with a history of a cervical procedure (i.e. ablation or cryotherapy); previous gynecological surgeries such as electrosurgical loop excision, cold knife conization, or hysterectomy; a history of pelvis radiotherapy; nondiagnostic or inadequate sampling; incomplete information; pregnancy; or known human immunodeficiency virus infection were excluded from participating. A subgroup of patients with TZ3 lesions was selected for analysis.

Demographics and clinical characteristics for all eligible women were collected and included age, gravidity, parity, menopausal status, cytologies, HPV status, colposcopic impressions, and lesion size. The study was approved by the Institutional Review Board of the Chinese Academy of Medical Sciences and Peking Union Medical College (approval number, CAMS & PUMC-IEC-2022–022) and performed in accordance with the tenets of the Declaration of Helsinki. The requirement for written informed consent was waived because this was a retrospective observational study and data were anonymized.

### Cytology and HPV testing

Cytology was performed using the liquid-based ThinPrep test. In brief, this test is performed by introducing a cell brush into the external cavity and scraping cells from the exocervix and endocervix. The cells are then placed on a smear slide and fixed. Cytology results were classified into one of the following five categories, according to the Bethesda System [13]: NILM, ASC-US (atypical squamous cells of unknown significance), LSIL (low-grade squamous intraepithelial lesion), ASC-H (atypical squamous cells, cannot exclude high-grade squamous intraepithelial lesion), or HSIL+ (high-grade squamous intraepithelial lesion and/or squamous cervical carcinoma). HPV status is defined as HPV 16/18, non-16/18 HR-HPV, or negative. HPV tests method are not described in detail because this was not deemed pertinent to this study.

### Colposcopy/ECC and biopsy procedures

Colposcopy was used to check for tiny lesions on the superficial layer of the cervix that cannot be seen by the naked eye. A digital colposcope was used to enlarge the vagina mucosa and cervix. Any changes in its surface morphology and terminal vascular network can be was digitally processed. Colposcopic examination was used to assess TZ type of (i.e. TZ1, visible; TZ2, partially visible; or TZ3, not visible) and to obtain a colposcopic impression (normal/benign, low-grade, or high-grade). Lesion area was categorized according to size as < 1/3, 1/3–2/3, or > 2/3.

All abnormalities detected on colposcopy were biopsied directly. If necessary, ECC was performed after cervical biopsy using a Kevorkian curette. ECC and biopsy results were classified as normal, LSIL, HSIL, or invasive cancer according to the Lower Anogenital Squamous Terminology system [14]. The worst grade lesion present was considered the final diagnosis. HSIL+ cases included HSIL and invasive cancers and the remainder were <HSIL. Pathological diagnosis based on ECC was reviewed by two experienced local pathologists working independently, with any disagreements were resolved through discussion.

### Statistical analysis

Supplement 1 outlines hows quantitative and categorical variables were dealt with and how coding was conducted for each group. Accuracy was calculated as the overall consistency of histological diagnosis between biopsy and ECC. HSIL+ diagnostic yield by biopsy was defined as HSIL+ cases that would have been detected by cervical biopsy but missed by ECC alone. HSIL+ diagnostic yield by biopsy and ECC was defined as HSIL+ cases that would have been detected by ECC and cervical biopsy. HSIL+ diagnostic yield by ECC was defined as HSIL+ cases that would have been detected by ECC but missed by cervical biopsy alone. Univariate and multivariate analyses were performed using standard chi-squared tests and logistic regression with an enter approach to assess independent risk factors for HSIL+, which are reported as odds ratios (ORs) with 95% confidence intervals (CIs).

Values were stratified for each of these five indicators, according to risk factors identified. Group distributions are represented and compared with histograms. Statistical analysis was performed using SPSS software (version 25.0) and EXCEL (version 2016). All *p*-values were two-sided with the threshold for statistical significance set at 0.05.

## Results

### Study population and data characteristics

**Fig. 1:**
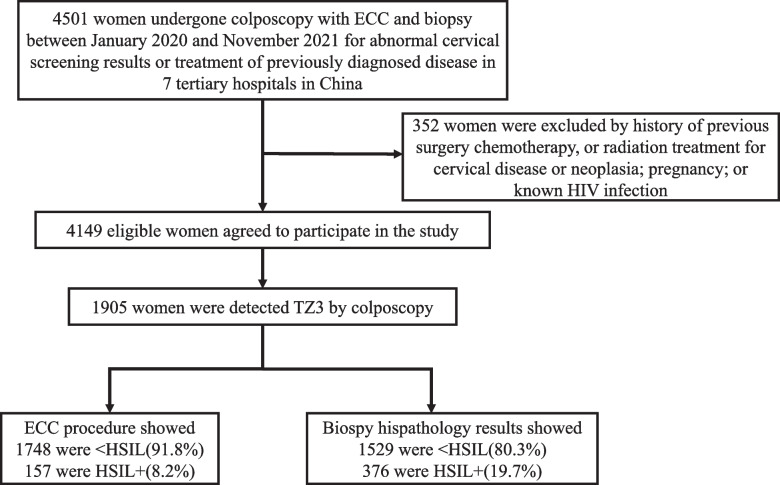
Flow chart of the participants inclusion and exclusion

Data from 4,501 patients with complete, detailed ECC and biopsy records were initially considered. 352 were excluded due to incomplete information. 1,905 eligible women with TZ3 lesions were included and analysed according to our predefined selection criteria. Among ECC diagnosis, 91.8% (*n* = 1,748) were <HSIL and 8.2% (*n* = 157) were HSIL+ . Among biopsy diagnosis, 80.3% (*n* = 1,529) were <HSIL and 19.7% (*n* = 376) were HSIL+ (Fig. 1).

Table 1 provides demographic and clinical characteristics for participants. 71.9% (*n* = 1,369) of the women had 1–3 pregnancies. 80.1% (*n* = 1,527) had given birth once or twice, and 70.3% (*n* = 1,340) were considered premenopausal. The most common cytological results were NILM (32.5%; *n* = 619) and AS-CUS (34.3%; *n* = 654). Additionally, 33.9% (*n* = 646) of this sample had HPV 16/18 infections and 57.7% (*n* = 1,099) had other types of HPV infection. The colposcopic impression was high-grade in 18.5% (*n* = 353) and low-grade in 61.5% (*n* = 1,171). Most of this sample i.e. 70.9% (*n* = 1,351) were in the 1/3–2/3 lesion area subgroups.

**Table 1:** Demographics and clinical characteristics of study population

Characteristics	N	%
**Total**	1905	100
**Age** (years)		
< 30	152	8.0
30–39	536	28.1
40–49	586	30.8
> 50	631	33.1
**Gravidity**		
0	100	5.2
1–3	1369	71.9
> 3	436	22.9
**Parity**		
0	173	9.1
1–2	1527	80.1
> 2	205	10.8
**Menopause**		
No	1340	70.3
Yes	565	29.7
**Cytology**		
NILM	619	32.5
ASC-US	654	34.3
LSIL	336	17.7
ASC-H	120	6.3
HSIL	176	9.2
**HPV status**		
Negative	160	8.4
Non-16/18 hrHPV	1099	57.7
HPV16/18	646	33.9
**Colposcopic impressions**		
Normal/benign	381	20.0
Low-grade	1171	61.5
High-grade	353	18.5
**Size of lesion**		
< 1/3	460	24.2
1/3–2/3	1351	70.9
> 2/3	94	4.9
**ECC**		
<HSIL	1748	91.8
HSIL+	157	8.2
**Biopsy**		
<HSIL	1529	80.3
HSIL+	376	19.7

Abbreviations: *NILM* negative for intraepithelial lesion or malignancy, *ASC-US* atypical squamous cells of undetermined significance, *LSIL* low-grade squamous intraepithelial lesion, *ASC-H* atypical squamous cells which did not exclude high grade squamous intraepithelial lesion, *HSIL* high-grade squamous intraepithelial lesion, *hr-HPV* high-risk human papillomavirus, <*HSIL included normal and LSIL* HSIL+ included HSIL and invasive cancers, *ECC* endocervical curettage

### HSIL + diagnosis with biopsy and ECC

Table 2 compares pathological results for ECC and lesion-directed biopsies. In all women with TZ3 lesions, 7.4% (*n* = 142) HSIL+ cases were detected by both ECC and biopsy. 12.3% (*n* = 234) HSIL+ cases detected by biopsy but missed by ECC alone. 20.5% (*n* = 391) HSIL+ cases were detected by ECC and biopsy totally. The 0.8% (*n* = 15) of HSIL+ cases were missed by biopsy alone but were detected when biopsy was applied with ECC.

**Table 2:** The performance of ECC and biopsy for detecting HSIL+

Histopathology diagnosis by ECC	Biopsy Histopathology diagnosis		
	<HSIL	HSIL+	Total
<HSIL	1514 (79.5%)	234 (12.3%)	1748 (91.8%)
HSIL+	15 (0.8%)	142 (7.4%)	157 (8.2%)
Total	1529 (80.3%)	376 (19.7%)	1905 (100%)
	^a^HSIL+ diagnostic yield by biopsy 234/1905 = 12.3% ^b^HSIL+ diagnostic yield by both biopsy and ECC 142/10905 = 7.4% ^c^HSIL+ diagnostic yield by ECC 15/1905 = 0.8% ^d^HSIL+ diagnostic yield totally (376 + 157–142) /1905 = 20.5%		

Abbreviation: *ECC* endocervical curettage, <*HSIL included normal and LSIL* HSIL+ included HSIL and invasive cancers

^a^HSIL+ diagnostic yield by biopsy: HSIL+ cases that would have been detected by biopsy but missed by ECC alone

^b^HSIL+ diagnostic yield by both biopsy and ECC: Cases that ECC and biopsy results are HSIL+

^c^HSIL+ diagnostic yield by ECC: HSIL+ cases that would have been detected by ECC but missed by biopsy alone

^d^HSIL+ diagnostic yield totally: HSIL+ cases that would have been detected by ECC and biopsy

### Risk factors for HSIL + detected by ECC

The women diagnosed HSIL+ by ECC were stratified by age, cytology, HPV status, colposcopic impression, and lesion area (Table 3) for identification of risk factors. Significant differences in growth trends were found across subgroups (*p* < 0.05). Logistic regression analysis showed that the risk of detection of HSIL+ was higher in women aged 40–49 years (OR 2.653, 95% CI 1.009–6.977) and those older than 50 years (OR 2.545, 95% CI 0.965–6.716) than in those aged younger than 30 years. There was a higher risk of ASC-H cytology (OR 2.415, 95% CI 1.213–4.808) and HSIL cytology (OR 2.933, 95% CI 1.648–5.220) than of NILM. Women with HPV 16/18 infection were at higher risk of HSIL+ than those who were HPV 16/18-negative (OR 2.299, 95% CI 0.942–5.613). Patients with a high-grade colposcopic impression were more likely to be diagnosed with a precancerous lesion than those with a normal colposcopic impression (OR 35.884, 95% CI 12.214–105.426).

**Table 3:** Risk factors of ECC in detecting HSIL+

**Subgroups**	**ECC diagnosis**		**Univariate**		**Multivariate**	
	HSIL+ (*n* = 157)	<HSIL (*n* = 1748)	OR (95%CI)	*P*	OR (95%CI)	*P*
**Age** (years)						
< 30	6	146			Reference	––
30–39	36	500	1.752 (0.724–4.240)	0.214	1.539 (0.574–4.125)	0.392
40–49	56	530	2.571 (1.086–6.086)	0.032	2.653 (1.009–6.977)	**0.048**
> 50	59	572	2.510 (1.063–5.927)	0.036	2.545 (0.965–6.716)	0.059
**Cytology**						
NILM	30	589			Reference	––
ASC-US	23	631	0.716 (0.411–1.246)	0.237	0.897 (0.483–1.665)	0.340
LSIL	11	325	0.665 (0.329–1.344)	0.255	0.839 (0.388–1.816)	0.835
ASC-H	26	94	5.430 (3.076–9.588)	0.000	2.415 (1.213–4.808)	**0.011**
HSIL	67	109	12.068 (7.493–19.437)	0.000	2.933 (1.648–5.220)	**0.000**
**HPV status**						
Negative	7	153			Reference	––
Non-16/18 hrHPV	48	1051	0.998 (0.444–2.246)	0.997	0.898 (0.362–2.229)	0.743
HPV16/18	102	544	4.098 (1.866–8.999)	0.000	2.299 (0.942–5.613)	**0.001**
**Colposcopic impressions**						
Normal/benign	4	377			Reference	––
Low-grade	24	1147	1.972 (0.680–5.720)	0.211	2.523 (0.838–7.598)	0.064
High-grade	129	224	54.278 (19.795–148.832)	0.000	35.884 (12.214–105.426)	**0.000**
**Size of lesion**						
< 1/3	32	428			Reference	––
1/3–2/3	104	1247	1.115 (0.739–1.683)	0.602	0.557 (0.328–0.947)	0.378
> 2/3	21	73	3.848 (2.104–7.037)	0.000	1.249 (0.567–2.751)	0.072

Abbreviation: *ECC* endocervical curettage, *OR* odds ratio, *NILM* negative for intraepithelial lesion or malignancy, *ASC-US* atypical squamous cells of undetermined significance, *LSIL* low-grade squamous intraepithelial lesion, *ASC-H* atypical squamous cells which did not exclude high grade squamous intraepithelial lesion, *HSIL* high-grade squamous intraepithelial lesion, *hr-HPV* high-risk human papillomavirus

### Stratification of HSIL+ diagnostic yield by ECC only

**Fig. 2:**
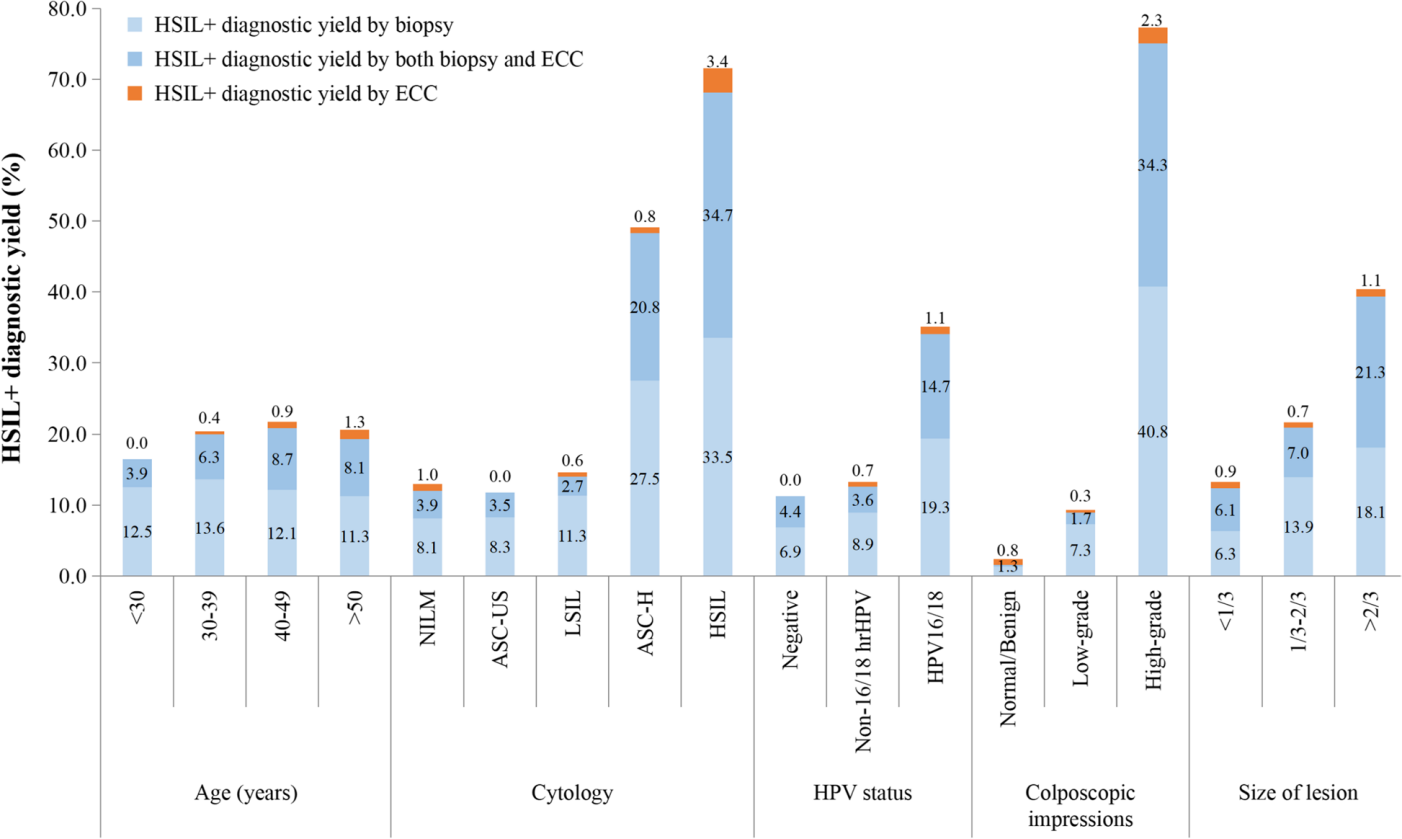
Stratified HSIL+ diagnostic yield. The results for HSIL+ detected by ECC and biopsy when stratified by age group, cytology, HPV status, colposcopic impression, and lesion area. Abbreviation: ASC-US: atypical squamous cells of undetermined significance; ASC-H: atypical squamous cells that cannot exclude high grade squamous intraepithelial lesion; ECC: endocervical curettage; HSIL: high-grade squamous intraepithelial lesion; HSIL+ : high-grade squamous intraepithelial lesions or worse; <HSIL included normal and low-grade squamous intraepithelial lesion; HPV: human papillomavirus; LSIL: low-grade squamous intraepithelial lesion; NILM: negative for intraepithelial lesions or malignancy; TZ: transformation zone; HSIL + diagnostic yield by biopsy: HSIL+ cases that would have been detected by biopsy but missed by ECC alone. HSIL+ diagnostic yield by both biopsy and ECC: Cases that ECC and biopsy results are HSIL+. HSIL+ diagnostic yield by ECC: HSIL+ cases that would have been detected by ECC but missed by biopsy alone. HSIL+ diagnostic yield totally: HSIL+ cases that would have been detected by ECC and biopsy

Figure 2 shows the results for HSIL+ detected by ECC and biopsy when stratified by age group, cytology, HPV status, colposcopic impression, and lesion area. ECC detected HSIL+ in 0.8% (*n* = 15) of the 1,905 cases. This means, 125 ECCs needed to be performed to identify one case of HSIL+ that would not have been identified by colposcopically directed biopsy. However, ECC based HSIL+ diagnostics yield rates ranging from 0.0% to 3.4% in various risk subgroups. In the following risk subgroups, ECC can benefit more people. The rate of HSIL+ diagnosed by ECC alone increased to 1.3% (8/631) in women over 50 years of age. The highest yield of HSIL+ from ECC was observed in women with HSIL cytology of 3.4% (6/176) and high-grade impression of 2.3% (8/353). The HSIL+ diagnostic yield by ECC in women with HPV16/18 infection was 1.1% (7/646), and in non-16/18 hrHPV group, 0.7% (8/1099) additional HSIL+ cases were detected by ECC. ECC procedure done in the lesion area > 2/3 population detected up to 1.1% (1/94) of HSIL+ cases which we also missed when using biopsy alone.

## Discussion

ECC is often used as an adjunct to biopsy to diagnose HSIL+ in women with TZ3 lesions who have cervical canal atrophy, and sometimes to detect occult lesions that are difficult to observe with a colposcope. However, the value of ECC as a complementary test in the clinical setting has been controversial. The purpose of this study was to identify women with TZ3 lesions who would benefit most from ECC for HSIL+ and to assess the advantages of this test. Analysis of data from 1,905 women whose ECC and biopsy information was clear revealed that routine ECC following biopsy detected 20.5% of the HSIL+ cases. The HSIL+ diagnostic yield associated with ECC was 0.8% which suggests that to detect one additional HSIL+ case, missed by biopsy, would require screening of 125 women. This means, many women would be subjected to the protracted pain and discomfort associated with unnecessary ECC procedures. Therefore, it would be unwise to perform ECC in all women with abnormal cytology or HPV test results, and a high-risk group should be selected.

Stratified results suggest that age, cytology, HPV status and colposcopic impressions are risk factors for HSIL+ detection with ECC. HSIL cytology, HPV 16/18 infections, and a high-grade colposcopic impression favourably impacted the detection rate in middle-aged and older women. Specifically, we found that ECC detection of HSIL+ was more likely in women over 50 years of age and those aged 40–49 years compared to those younger than 30 years. This finding reflects the age-related decrease in hormone levels and atrophy of the cervical canal to the point where part of all of the TZs become invisible. This is consistent with the findings of Schneider et al. [15] and Shepherd et al. [16], who found that ECC was of most benefit to women older than 50 years, in terms of decreasing the incidence of cervical cancer and mortality. In the latest standarizing colposcopy guidelines, it is suggested to American practitioners that all patients over 40 years old should initially choose cervical curettage [8]. However, there is still no united view of the cut-off age and studies of more varied ethnicities are required to understand this.

In our study, the cytological subgroups of HSIL and ASC-H were the recommended group for doing the ECC procedure compared to normal cytological women. Poomtavorn et al. also concluded that ECC should not be performed in women with ASC-US or LSIL in view of the extremely small risk of HSIL+ [17]. In our study, Multivariate analysis revealed that the probability of diagnosing HSIL+ with ECC was approximately twice as much in women who were HPV 16/18-positive compared to those who were HPV 16/18-negative. The risk for those with non-16/18 but high-risk HPV cases was 0.898. HPV 16/18 infection has been found in 70% of women with invasive precancerous lesions [18, 19]; however, 13 HPV subtypes are carcinogenic when non-16/18 high-risk HPV is included [20, 21]. We also found that seven of 160 patients who were HPV-negative had HSIL according to ECC. 11 of the 234 with HSIL detected by biopsy, were HPV-negative. This evidence supports the notion that HPV detection alone increases the number of missed diagnoses. Bogani et al.'s research illustrates the universality of high-grade cervical lesion in 15% of high-risk-HPV-negative patients after conization [22].

Colposcopic impressions revealed HSIL+ in 36 times as many instances in severely ill women compared to healthy individuals. These findings suggest that when screening a large group of women, cytological AS-CUS, negative HPV status, and a low-grade colposcopic impressions for women younger than 30 years of age may help prevent unnecessary flesh contusions. Furthermore, they suggest a need to incorporate these prognostic factors into a convenient risk assessment tool for accurate, precise, and standardized quantitative clinical decision-making. The five-factor nomogram developed by Li et al. was found to have a high degree of discrimination and calibration and performed well in terms of utility in an internal and external validation set of 2,088 patients [23].

In previously reported studies, the rate of detection of HSIL+ by ECC has ranged widely from 1.1% to 18.5% [4, 5, 24]. In our present study, the additional detection rate was 0.8%, which is comparable with the figure of 0.6% in another Chinese study [25]. The variable detection rates could reflect use of different study endpoints and patient populations. The majority of the women in our study visited a clinic rather than a screening facility, and the characteristics of CIN2 lesions, are frequently constrained, small, and have low reproducibility, making a diagnosis uncertain and challenging. Given this low homogeneity restriction, integrating techniques into ECC may be a viable option to bring clarity to diagnostic classification. Shah et al. applied p16/Ki67 dual stain to 58 ECC specimens and diagnosed 18 additional cases of HSIL [26]. Maximiliano et al. attempted to overcome the drawbacks associated with the sparseness of ECC tissue using cell concentration methods, which may be a suitable strategy for qualitative improvement of the sensitivity of ECC and its diagnostic value [27]. Furthermore, Rubesa-Mihaljevic et al. found ECC sensitivity was higher for samples with abundant materials compared to samples with relatively few [28]. Uses of inexpensive and less painful sampling instruments to increase the rate of satisfactory specimen and reduce the reliance on senior physicians are additional ways of increasing patients’ compliance with procedures and improving lesion detection rates [29]. These measures will not only help to increase the accuracy of ECC but also make it possible for clinical examinations to be effectively integrated in low-income and middle-income countries with limited resources, striking a balance between the costs of clinical examinations and the availability of experienced practitioners.

Without high-risk population screening, only 0.8% (15/1905) of the women in the current study had TZ3 lesions, which resulted in an unsatisfactory overall gain. However, this figure increased to 3.4% in the subgroup with cytological HSIL and to 2.3% in the subgroup with low-grade colposcopy impressions. Following a Chinese study, only four additional cases of HSIL+ were detected by ECC, all of whom were in patients aged at least 40 years and with TZ3 lesions [30]. Gage et al. reported an additional detection rate of 5.4% (132/2433) in all cases with CIN2+ pathology when ECC was used and observed that the less visible the TZ, the higher the additional cytology detection rate [4]. This suggests that colposcopists should concentrate on older but still accurate colposcopy impressions when assessing patients with TZ3 lesions. A supplemental ECC method should therefore be used to clarify diagnosis if lesion level in the visible area is not compatible with the risk assessment based on screening findings. This is also in line with the most recent American Society of Colposcopy and Cervical Pathology recommendations, which state that ECC is preferable for non-pregnant women in whom colposcopy is insufficient and there is a slight risk of morbidity but no obvious lesion [31].

In addition to use of ECC for diagnosis, some guidelines suggest large loop excision of the transformation zone (LLETZ) in patients with TZ3 and abnormal cytological results to remove all the transformed areas (not just the diseased portions) and the cervical canal with sufficient length to ensure that there is no diseased tissue within at least 2–3 mm from the cutting edge and remove all the recesses in the transformed areas to reduce the risk of missed diagnosis [31]. A previous study found that diagnostic LLETZ detected six cases of CIN2+ in 40 patients with persistent HPV infection and normal cytology that were not found by biopsy, giving a detection rate of 15% [32]. Another study reported that 25% (*n* = 6) of 24 women with HPV-positive/normal cytology who underwent diagnostic conization for TZ3 had CIN2+ [33]. Moreover, preoperative diagnosis by conization protects against recurrence after radical hysterectomy [34, 35]. It is noteworthy that an ECC abnormality is an independent risk factor for residual disease after LLETZ [35, 36]. However, the latest guidelines stipulate that if a subsequent resection is planned, the sampling device should not be placed in the cervical canal [8]. Therefore, attention should be paid to the results of ECC, follow-up monitoring should be strengthened, and risk factors should be considered in the selection of individualized diagnostic and treatment methods, which may play an active role in cervical intraepithelial neoplasia and residual tumors after surgery.

This study's biggest strength is that it adds to the evidence regarding the value of ECC for women with TZ3 lesion, patients since they had the highest rate of HSIL+ discovery. Our results in a large research population drawn from seven provinces in China underscore the abuse risk of ECC. However, the study has some limitations. First, this study was retrospective so the possibility of bias in the data cannot be excluded. Second, the majority of the study population in this study was middle-aged and older women with TZ3 lesion, with only a small number of young women in the specific population.

## Conclusion

This study was performed in an effort to improve the rate of detection of HSIL+ in women with TZ3 lesions without subjecting these women to unnecessary discomfort. We identified high-risk groups in whom ECC is warranted, namely, middle-aged and elderly women with a high-grade colposcopic impression, high-grade cytology, and HPV 16/18 infection. These findings may reduce the number of missed occult HSIL+ cases and adds to the evidence base related to the use of ECC in clinical practice. 

## Supplementary Information

**Additional file 1:**

## Data Availability

The datasets generated and/or analyzed during the current study are not publicly available due personal information protection, patient privacy regulation, and medical institutional data regulatory policies, etc., but are available from the corresponding author on reasonable request and with permission of the Chinese Academy of Medical Sciences and Peking Union Medical College data sharing committee.

## Abbreviations

ASC-US: Atypical squamous cells of undetermined significance
ASC-H: Atypical squamous cells that cannot exclude high grade squamous intraepithelial lesion
CI: Confidence intervals
CAMS & PUMC: Chinese Academy of Medical Sciences and Peking Union Medical College
ECC: Endocervical curettage
HSIL: High-grade squamous intraepithelial lesion
HSIL+: High-grade squamous intraepithelial lesions or worse; <HSIL included normal and low-grade squamous intraepithelial lesion
HPV: Human papillomavirus
IRB: Institutional review board
LSIL: Low-grade squamous intraepithelial lesion
LAST: Lower Anogenital Squamous Terminology
NILM: Negative for intraepithelial lesions or malignancy
OR: Odds ratio
TZ: Transformation zone
